# Factors affecting cervical cancer knowledge among women in Addis Ababa, Ethiopia: A population-based study

**DOI:** 10.1371/journal.pgph.0004961

**Published:** 2025-07-28

**Authors:** Ebrahim Mohammed Gebaba, Girma Taye Aweke, Adamu Addissie, Mathewos Assefa, Ahmedin Jemal

**Affiliations:** 1 Department of Reproductive, Family and Population Health, School of Public Health, Addis Ababa University, Addis Ababa, Ethiopia; 2 Department of Epidemiology and Biostatistics, School of Public Health, Addis Ababa University, Addis Ababa, Ethiopia; 3 Department of Oncology, School of Medicine, Addis Ababa University, Addis Ababa, Ethiopia; 4 Surveillance and Health Equity Science, American Cancer Society, Atlanta, Georgia, United States of America; African Union, ETHIOPIA

## Abstract

Cervical cancer (CCa) is the second leading cause of cancer-related death among women in Addis Ababa and other regions of Ethiopia. The aim of this study was to assess the level of awareness, knowledge, and knowledge of predictors of CCa and screening among women aged 30–49 years in Addis Ababa, Ethiopia. A total of 1980 women aged 30–49 were randomly selected through multistage sampling in Addis Ababa. Data were collected using the Kobo Toolbox after developing and validating the data collection tools. Data were analyzed using SPSS version 26. Logistic regression was used to identify predictors of cervical cancer awareness and knowledge, with a significance level set at a P-value of <0.05. Out of 1,890, 1,881 (99.5%) responded, 1,736 (92.3%) had heard of CCa, 1,015 (54.0%) were aware that Human Papillomavirus (HPV) infection is a risk factor, and 1,237 (65.8%) knew that HPV vaccination prevents CCa. More than half, 1,025 (54.5%) (95%CI = 52.2%–56.8%) had good knowledge of risk factors, 980 (52.1%) (95% CI = 49.8, 54.4%) had poor knowledge of symptoms, and 963 (51.2%) (95% CI = 48.9%, 53.5%) exhibited poor awareness. Overall, 990 (52.6%) (95% CI = 50.4, 54.9%) participants had good knowledge of cervical cancer. Factors such as higher educational attainment (AOR = 2.23; 95% CI: 1.49, 3.34), higher family income (AOR = 1.50; 95%CI: 1.01, 2.23), knowing health facility offering screening (AOR = 1.34; 95% CI: 1.07, 1.68), and knowing someone having cervical cancer (AOR = 5.46; 95% CI: 3.31, 8.97) were significantly associated with overall knowledge levels. Awareness and knowledge about cervical cancer and screening are suboptimal. These findings underscore the need for educational intervention to enhance awareness and knowledge of cervical cancer to reduce the high burden of the disease in the city and other regions of Ethiopia.

## 1. Introduction

Cervical cancer is the second leading cause of morbidity and cancer related deaths among women in Addis Ababa and other parts of Ethiopia [1,2]. In Ethiopia, 8,168 new cases of cervical cancer are registered in 2023, indicating an incidence of 22.3 per 100,000 at standardized age. During the same year, 5,975 women died from the disease, resulting in an age-standardized mortality rate of 16.8 per 100,000 women. Without intervention, it is projected that total of 191,876 women in Ethiopia will die from cervical cancer between 2020 and 2070 [3,4]. Nearly two-thirds of the cases in Addis Ababa and other parts of the country are diagnosed at an advanced stage of the disease [5,6] when treatment is less effective and the chance of survival is poor [6].This is due to the low uptake of early screening and detection [7]. The actual number of cases occurring in Addis Ababa, is likely underestimated because the registry does not collect data from all hospitals and diagnostic facilities in the city, additionally, many patients seek traditional rather than conventional treatment, leading to under diagnosis [8].

In low-middle income countries (LMICs), where resources are scarce and available treatment choices are sometimes inaccessible and expensive, cervical cancer continues to be a major worldwide burden and a major therapeutic challenge [9]. Therefore, it is crucial for all nations to endorse the 2020 World Health Assembly resolution calling for the “Elimination of Cervical Cancer” by 2030, with the following three goals: vaccinating 90% of girls against human papilloma virus HPV by age 15, screening 70% of women with high-performance tests at ages 35 and 45, and treating 90% of precancerous lesions and managing 90% of invasive cancer cases [10]. Three-fourth (75%) of the target adolescent girls (Ages 9–14 Years) were received second dose against HPV in Ethiopia and nearly 4% of target women were screened [11]. Following the World Health Organization’s cervical cancer elimination strategies, the Ethiopian government launched guidelines for cervical cancer elimination, focusing on raising awareness and knowledge among target women and the community as well as providing screening services and treatment [12].

Many studies have reported that most women have low awareness and knowledge of cervical cancer. Studies from Addis Ababa showed that 43–76% of women had heard of cervical cancer screening (9–11). Less than one-third [13] of women were knowledgeable about cervical cancer. Studies from other parts of Ethiopia, such as Butajera, indicated that only 36% of women were aware of cervical cancer and less than 5% knew the symptoms of cervical cancer [14]. In Assosa, 53.5% of the participants demonstrated good knowledge of cervical cancer [15]. Studies involving women visiting health institutions for various services also showed a low level of awareness and knowledge. For example, among women visiting primary health care centers for antenatal follow-up, family planning, or postnatal care, 42.7% had heard of cervical cancer screening and 27.7% had adequate knowledge of cervical cancer screening [15]. Similarly, a community-based cross-sectional study of parents or guardians of daughters aged 9–17 years in Akaki-Kalty sub-city reported that 41.7% and 72.0% of women had good knowledge of cervical cancer and HPV, respectively [16], while 27.0% of women had never heard of the HPV vaccine [16]. However, these studies were limited because they were based on clinical series, small geographic areas, or women living with HIV, and their findings cannot be generalized to all women in Addis Ababa.

In this study, we examined the knowledge and awareness of cervical cancer risk factors and preventive measures among women aged 30–49 in Addis Ababa, based on data from a city-wide representative survey. We focused on this age group because they are recommended to undergo cervical cancer screening by the WHO and may be raising children who are eligible for the HPV vaccine. The aim of this study was to assess the awareness, knowledge and factors affecting knowledge of cervical cancer and screening.

## 2. Materials and methods

### 2.1. Ethical statement

Ethical clearance was obtained from the Institutional Ethics Review Board of Addis Ababa University, College of Health Sciences (IRB No. 081/22/SPH). Official letters were sent to the health centers by the Addis Ababa City Health Bureau. Permission was obtained from all participating health facilities. Each respondent was informed about the purpose and scope of the study. Verbal informed consent, as approved by the Institutional Review Board, was obtained from all participants prior to their enrollment. Data collectors recorded participants’ consent by marking a checklist provided on the consent form.

### 2.2. Study setting and population

Addis Ababa, the capital city of Ethiopia, has a population of over 5.7 million [17]. The city is divided into 11 administrative sub-cities, comprising 111 districts and 5,536 enumeration areas (EAs). There are 99 public primary care health centers and 12 public hospitals in Addis Ababa. This study included women aged 30–49 who had lived in Addis Ababa for more than six months and had no history of hysterectomy. It is estimated that nearly 17% of women are of reproductive age, and more than one-third 40% of women are between 30 and 49 years old [18]. Cervical cancer screening services are provided in all public health institutions.

### 2.3. Study design and sampling techniques

A population-based cross-sectional study design was used. The sample size was determined using the WHO-STEP sample size determination formula for non-communicable diseases (NCDs) [19], incorporating a design effect of 2.0, an age-sex estimation of 2 for females only, an 80% response rate, and a 5% margin of error. The proportion of 56.3% lacking knowledge about cervical cancer screening in Addis Ababa, as reported by Getachew et al. (2019) [15], was taken into account. Based on these assumptions, a sample size of 1890 was calculated. The number of enumeration areas (EA) or clusters (K) was determined by dividing the sample size (1890) by the number of households (30 HHs) to be interviewed per cluster. Therefore, the number of clusters (K) was calculated as 1890/30 = 63. Each cluster represents an EA.

Multistage sampling techniques were employed in this study. In the first stage, the city was clustered into 11 sub-cities. In the second stage, 63 EAs were proportionally allocated to the sub-cities using computer-based proportional allocation of the sample size. In the third stage, 30 HHs were randomly selected for each EA. Each EA was delineated from four directions (North, South, East, and West), and households were numbered and counted. The interval was calculated by dividing the number of households by 30 (K = No. of HHs/30). The k^th^ interval varied among EAs based on household size. If no target women were found in the selected HHs, the immediate adjacent HHs were chosen and the interval continued. In cases where there are more than one target women in the HHs unit, one woman was randomly selected by the data collector. Three repeated visits were conducted to include all the randomly selected women during the interviews. Out of the 1890 women selected to participate in the study, eight could not be traced, and one declined to participate.

**Fig 1:**
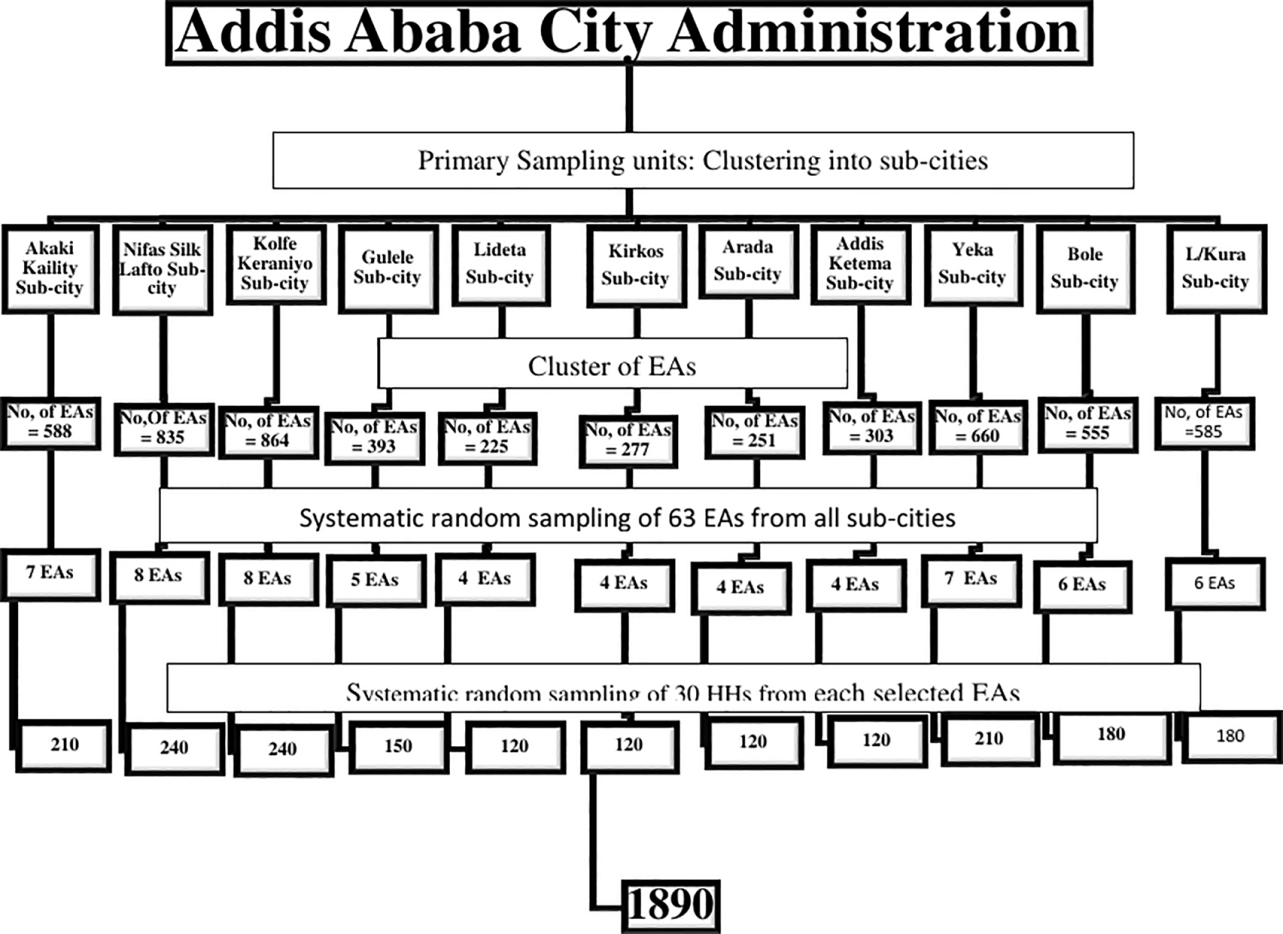
Sampling techniques flow chart for study conducted among women in Addis Ababa.

The study included women aged 30–49 years following the WHO’s recommendation for cervical cancer screening in low-income countries using the “See and Treat” approach [10]. Women under 30 years and over 49 years were excluded as they were not eligible for cervical cancer screening in the general population [10]. Pregnant women and those within three months postpartum were also excluded based on Ethiopian guidelines for cervical cancer prevention and control [20]. Women with a history of total hysterectomy were also excluded from the study (See Fig 1).

Fig 1 illustrates the sampling techniques and outlines the sampling procedures. First, Addis Ababa was divided into 11 sub-cities, with the enumeration areas allocated proportionally based on size. Thirty households were randomly selected from each enumeration area.

### 2.4. Variables

**Outcome variable:** knowledge level (Coded as overall knowledge level; Poor/Good)

**Independent variables**: it includes socio-demographic variables such as age, educational level, current occupational and marital status, partner`s educational status and average family monthly income. Variables for assessing awareness level includes hearing about cervical cancer and screening, knowing someone contracting cervical cancer, knowing cervical cancer screening availability and frequencies of screening, knowing health institution offering screening knowing about HPV vaccination and prevention methods of cervical cancer. Variables for knowledge of risk factors include knowing about HPV infection, HIV and other sexually transmitted infections (STIs), multiple partners, non-condom use, high parity, long term uses of contraceptives, initiation of sex at young age, and smoking. Variables for symptoms of the disease, such as vaginal bleeding between menstrual periods, a persistent smelly vaginal discharge, discomfort or pain during sex, longer and heavy menstrual periods, vaginal bleeding after menopause, persistent lower abdominal/pelvic pain, vaginal bleeding during or after sex, unexplained weight loss could be S/S of cervical cancer and vaginal itching were used to collect data

### 2.5. Data collection methods and analysis

Data were collected using structured and semi-structured face-to-face interviewer-administered questionnaires. The tool was adapted from the Cervical Cancer Awareness Measure (Cervical CAM) tool [21] and the African Women Awareness of Cancer (AWACAN) tool [22]. It was modified and validate through a literature review of studies conducted in various parts of the world, including Ethiopia [15,23–27]. The questionnaire was translated into the local language, Amharic, and then back-translated into English for consistency by language experts and public health researchers. The primary investigator trained nine data collectors and two supervisors for two days on the face-to-face interviews and pretest data collection procedures.

Tool validation involved three main steps: evaluation by a panel of 16 experts, translation into the local language, and internal reliability testing. The overall Content Validity ratio (CVR) and Item-Content Validity Index (I-CVI) scores were 0.68 and 0.88, respectively, indicating that the items were highly essential and relevant. The item-CVI for simplicity and clarity was 0.88 and 0.82, suggesting that the items were appropriately simple and clear. The lowest modified Kappa score was 0.49, showing moderate agreement, while the highest was 0.94, indicating perfect agreement among the experts. The overall Cronbach’s alpha was 0.79, demonstrating an acceptable level of reliability for the tool.

Data was collected using Kobo Toolbox software, which made all questions mandatory to prevent missing data, and were checked daily for completeness and consistency. The data was collected from April 01 to May 30, 2023.

Awareness and knowledge of cervical cancer symptoms were measured using 10 separate questions. Participants with values above the mean were considered to have good awareness and knowledge of cervical cancer symptoms. Knowledge of risk factors was assessed using 13 questions. Those scoring above the mean were categorized as having good knowledge of risk factors, while those scoring below the mean were categorized as having poor knowledge. The overall knowledge level of cervical cancer was assessed using the mean value of the knowledge of symptoms and risk factors. Participants scoring above the mean were considered to have good knowledge, whereas those scoring below the mean were considered to have poor knowledge of cervical cancer.

Data were checked for normal distribution using the Kolmogorov-Smirnov test (P < 0.001, indicating non-normal distribution), low skewness with Pearson’s coefficient of skewness (0.86 = indicating slight right-skew), and for multi-collinearity. Variance inflation factor was used (highest VIF was 1.623 for educational status) and goodness of fit assessed using adjusted R square (0.093).

Binary and multivariable logistic regression analyses were conducted using backward logistic regression, which excluded variables with no or small effects from the final model. Variables with P-values <0.25 during univariable logistic regression as well as important variables were selected for multivariable logistic regression to control for confounders [28,29]. The final model of multivariable logistic regression was used to declare the association of variables using adjusted odds ratios with 95% confidence intervals, and P-values <0.05 were used to indicate the association between independent and dependent variables. All statistical analyses were performed using SPSS version 26 (RRID: SCR_002865).

### 2.6. Ethical consideration

Ethical clearance was obtained from the Institutional Ethics Review Board of Addis Ababa University, College of Health Sciences (IRB No. 081/22/SPH). Official letters were sent to the health centers by the Addis Ababa City Health Bureau. Permission was obtained from all participating health facilities. Each respondent was informed about the purpose and scope of the study. Verbal informed consent, as approved by the Institutional Review Board, was obtained from all participants prior to their enrollment. Data collectors recorded participants’ consent by marking a checklist provided on the consent form.

## 3. Result

### 3.1. Socio-demographic characteristics

Almost all 1,881 participants (99.5%) responded to the survey. The median age of the participants was 35 years (IQR = 8). Regarding educational attainment of the study participants, 646 (34.3%) attained primary-level education. More than half 1064 (56.6%) of the participants were housewives (stay-at-home mothers), and 1,411 (75.0%) participants were married. Over one-third of the 727 (38.6%) of the participants had an average monthly income of 3,560 ETB, equivalent to US$ 65/month (See Table 1).

**Table 1:** Socio-demographic characteristics of study participants.

Variable	Frequency	Percent
**Age**		
30-39 years	1342	71.3
40-49 years	539	28.7
**Educational level**		
No formal education	343	18.2
Primary (1–8)	646	34.3
Secondary (9–12)	557	29.6
Diploma & above	335	17.8
**Current occupation**		
Housewife	1064	56.6
Gov. employee	207	11.0
self-employee	470	25.0
NGO (Non-govt. Institution)	124	6.6
Others*	16	0.9
**Current Marital status**		
Divorced	234	12.4
Married	1411	75.0
Single	136	7.2
Widowed	100	5.3
**Partner`s educational level**		
Unable to read and write	39	2.8
Read and write only	53	3.8
Primary (1–8)	384	27.2
Secondary (9–12)	516	36.6
Diploma and above	419	29.7
**Monthly Average family income**		
3560 ETB (US$65per month)	727	38.6
3561–6050ETB (US $65–110per month)	569	30.2
6051–11303 ETB (US $110–206 per month)	352	18.7
> 11303 ETB (US$ > 206 per month)	233	12.4

ETB = Ethiopian Birr, US = United State, * = working in shops as sales person, student etc

### 3.2. Awareness about cervical cancer and screening

Nine out of ten, 1736 (92.3%) of study participants had heard of cervical cancer and 1133 (60.2%) had heard of screening. Television (TV) was reported as a common source of information by 692 (39.9%) participants. Only 131 (7%) of the participants knew someone with a history of cervical cancer, and only 289 (15.4%) knew the types of cervical cancer screening methods. Of the 289 women who knew the types of cervical cancer screening methods (289), slightly more than one-third 108 (37.4%) knew visual inspection with acetic acid (VIA) as a screening method. Of those who reported knowing the frequency of screening (715), only 99 (13.8%) knew the recommended screening interval for VIA. More than half, 401 (56.1%) of study participants incorrectly reported that women should be screened annually. In terms of the prevention of cervical cancer, two-thirds, 1237 (65.8%) of women reported that HPV vaccination can prevent the chance of getting cervical cancer, and more than four-fifths, 1671 (88.8%) of the participants knew that early screening can prevent cervical cancer. Overall, 51.2% (95% CI = 48.9%, 53.5%) of the women had poor awareness of cervical cancer and cervical cancer screening (See Table 2).

**Table 2:** Awareness of cervical cancer, cervical cancer screening, and HPV vaccination.

Variables	Frequency	Percent
Have you ever heard of cervical cancer		
No	145	7.7
Yes	1736	92.3
Have you heard of cervical cancer screening		
No	748	39.8
Yes	1133	60.2
Information source for cervical cancer and screening		
Radio	32	1.8
TV	692	39.9
Health Professionals (HPs)	443	25.5
Relatives & Friends(RFs)	56	3.2
Health professionals and TV	371	21.4
All (Radio, TV, HPs and RFs)	125	7.2
Other	17	1.0
Know family members, friends or neighbors who have/had cervical cancer		
No	1750	93.0
Yes	131	7.0
Know types of cervical cancer screening methods		
No	1592	84.6
Yes	289	15.4
Type of cervical cancer screening methods you know		
HPV DNA test	44	15.2
VIA	108	37.4
Pap smear	49	17.0
HPV DNA test & Pap smear	28	9.7
HPV DNA test & VIA	26	9.0
VIA & Pap smear	26	9.0
VIA, HPV DNA & Pap smear	8	2.8
Know the frequency of cervical cancer screening with VIA		
No	1166	62.0
Yes	715	38.0
How frequent the women should be screened for cervical cancer with VIA?		
Once annually	401	56.1
Once every 2 years	124	17.3
Once every 3 years	99	13.8
Once every 4 years	7	1.0
Once every 5 years	84	11.7
Know health institution which provide cervical cancer screening in your residential area		
No	690	36.7
Yes	1191	63.3
Early cervical cancer screening reduce the chance of contracting cervical cancer		
No	60	3.2
Yes	1671	88.8
I don`t know	150	8.0
HPV vaccinations for Adolescent girls reduce chance of getting cervical cancer?		
No	69	3.7
Yes	1237	65.8
I don`t know	575	30.6
Cervical cancer can be transmitted from person to person		
No	1057	56.2
Yes	320	17.0
I don`t know	504	26.8
**Level of Awareness**		
Poor	963	51.2
Good	918	48.8

VIA= visual inspection with acetic acid

### 3.3. Knowledge of risk factors for cervical cancer

Table 3 describes the level of knowledge of the risk factors. Knowledge of risk factors was assessed using two methods: the first method involved open-ended questions, allowing women to answer or list the risk factors they know, and the second method used 13 questions each with a list of options for women to select from. Based on the 13 questions with response options of “no,” “yes” and “I don’t know,” more than half of them 1015 (54%) identified HPV as a risk factor. A total of 839 (44.6%) and 1465 (78.1%) women identified HIV infection and STIs as risk factors, respectively. More than four fifths of 1671 (88.8%) reported that unprotected sex increases the chance of contracting cervical cancer. The majority of women 1668 (88.7%) and 1454 (77.3%) reported that having many sexual partners and not undergoing regular cervical cancer screening increased their chances of developing cervical cancer. Nearly half (54.5%; 95%CI = 52.2%–56.8%) of the study participants demonstrated good knowledge of cervical cancer risk factors (See Table 3).

**Table 3:** Cervical cancer risk factors and preventive measures: Characteristics of study participants in Addis Ababa City.

Risk factors	No	Yes	I don`t know
Human Papilloma virus (HPV) infection causes cervical cancer?	191 (10.2%)	1015 (54.0%)	675 (35.9%)
HIV/AIDS infection causes cervical cancer	686 (36.5%)	839 (44.6%)	356 (18.9%)
Other STIs * causes cervical cancer	154 (8.2%)	1469 (78.1%)	258 (13.7%)
Birth control pill for > 5 years increase chance of developing cervical cancer	861 (45.8%)	504 (26.8%)	516 (27.4%)
Using condoms reduce chance of getting cervical cancer	351 (18.7%)	1214 (64.5%)	316 (16.8%)
Unprotected sex increases the chance of cervical cancer	71 (3.8%)	1671 (88.8%)	139 (7.4%)
Smoking cigarettes increase chance of developing cervical cancer	514 (27.3%)	1128 (60.0%)	239 (12.7%)
Sex at a young age increases chance of developing cervical cancer	253 (13.5%)	1453 (77.2%)	175 (9.3%)
Poor personal hygiene will increase chance of getting cervical cancer	68 (3.6%)	1760 (93.6%)	53 (2.8%)
Giving birth to >3 children increase chance of developing cervical cancer	229 (12.2%)	1436 (76.3)	216 (11.5%)
Having multiple sexual partners increases can the chance of developing cervical cancer	136 (7.2%)	1668 (88.7%)	77 (4.1%)
Not going for regular screening for cervical cancer increase chance of develop cervical cancer	281 (14.9%)	1454 (77.3%)	146 (7.6%)
Bewitched/witchcraft/evil spirits increase chance of developing cervical cancer	1651 (87.8%)	65 (3.5%)	165 (8.8%)

*STIs=Sexually transmitted infections, CCa = Cervical Cancer

**Fig 2:**
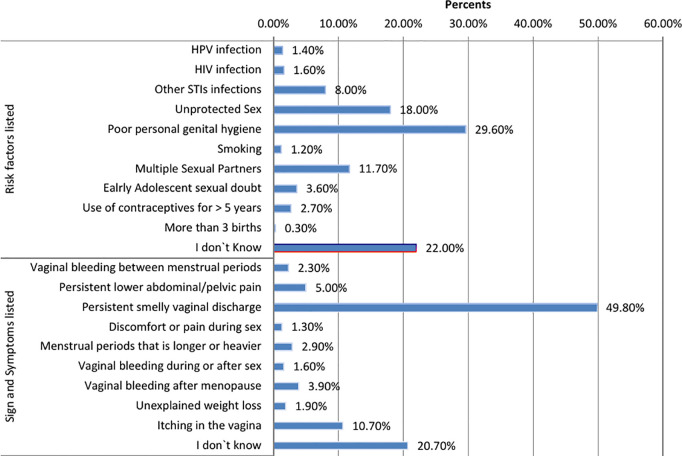
Risk factors and sign and symptoms participant women listed before getting any option of question about risk factors and sign and symptoms in Addis Ababa.

Fig 2 describes the responses to an open-ended question used to assess knowledge of risk factors. Based on the responses to the open-ended questions about risk factors for cervical cancer, the majority of women 556 (29.6%) identified poor personal hygiene as a risk factor, while only 26 (1.4%) identified HPV infection as a risk factor. One-fifth of the women 414 (22.0%) reported that they did not know of any risk factors.

### 3.4. Knowledge of signs and symptoms of cervical cancer

Knowledge of signs and symptoms was assessed using two methods. The first method involved open-ended questions, allowing women to list the signs and symptoms that they knew. The second method used 10 questions with response options “no,” “yes” and “I don’t know” for each question to select from. Based on this assessment method, 1707 (90.7%) reported that “persistent bad foul vaginal discharge” is a common symptom of cervical cancer. The second most common symptom identified by women was itching in the vagina, with 1549 (82.3%) acknowledging it. Nearly one in four participants 502 (26.7%) reported that vaginal bleeding between menstrual periods is a symptom of cervical cancer, while one-fifth of women 90 (20.7%) did not know any symptoms of cervical cancer. Nearly half (52.1%; 95% CI = 49.8, 54.4%) of the women had good knowledge of cervical cancer signs and symptoms, and nearly half of study participants (52.6%; 95% CI = 50.4, 54.9%) demonstrated good overall knowledge (See Table 4).

**Table 4:** Knowledge of the signs and symptoms of cervical cancer among study participants in Addis Ababa.

Sign and Symptoms	No	Yes	I don`t know
Vaginal bleeding between menstrual periods could be sign & symptoms of cervical cancer	1075 (57.2%)	502 (26.7%)	304 (16.2%)
A persistent smelly vaginal discharge could be sign & symptoms of cervical cancer	66 (3.5%)	1707 (90.7%)	108 (5.7%)
Discomfort or pain during sex could be sign & symptoms of cervical cancer	361 (19.2%)	1300 (69.1%)	220 (11.7%)
Menstrual periods longer or heavier than usual could be sign & symptoms of cervical cancer	457 (24.3%)	1186 (63.1%)	238 (12.7%)
Vaginal bleeding after menopause could be sign & symptoms of cervical cancer	423 (22.5%)	1170 (62.2%)	288 (15.3%)
Persistent lower abdominal/pelvic pain could be sign & symptoms of cervical cancer	514 (27.3%)	1045 (55.6%)	322 (17.1%)
Vaginal bleeding during or after sex could be sign & symptoms of cervical cancer	376 (20.0%)	1301 (69.2%)	204 (10.8%)
Unexplained weight loss could be S/S of cervical cancer?	726 (38.6%)	868 (46.1%)	278 (15.3%)
Itching in the vagina could be sign & symptoms of cervical cancer	213 (11.3%)	1549 (82.3%)	119 (6.3%)

Fig 2 shows the common symptoms reported by women in response to an open-ended question. Out of the total 939 (49.80%) study participants “persistent bad foul vaginal discharge” was mentioned as a common symptom, while 390 (20%) were unaware of any symptoms of cervical cancer. In both methods of listing and selecting options, study participants identified “persistent bad-foul vaginal discharge” as a common symptom (See Fig 2).

Fig 2 shows the risk factors and symptoms that were identified by study participants. This figure describes the risk factors and symptoms of cervical cancer as reported by the women during the interview without a list of options being provided.

### 3.5. Factors associated with overall knowledge of cervical cancer and screening

Table 5 describes the factors that showed a significant association with overall knowledge of cervical cancer and screening. Women with a diploma or higher education level had 2.23 times higher odds (AOR = 2.23; 95% CI: 1.49, 3.34) of having good overall knowledge of cervical cancer and screening than women with no formal education. As the average monthly income increased, the knowledge of women also increased. The odds of having good overall knowledge were 1.50 time higher (AOR = 1.50; 95%CI: 1.01, 2.23) among women with a monthly income of > 11303 ETB as compared to those with a monthly income of ≤ 3560 ETB. Knowledge of health facilities offering cervical cancer screening is also significantly associated with knowledge of cervical cancer and screening. The odds of having good overall knowledge were 1.34 times higher (AOR = 1.34; 95% CI: 1.07, 1.68) among women who knew health facilities providing cervical cancer screening. Likewise, the odds of having good overall knowledge of cervical cancer and screening were more than five times higher (AOR = 5.46; 95% CI: 3.31, 8.97) among women who knew someone with cervical cancer than among their counterparts (See Table 5).

**Table 5:** Factors associated with good overall knowledge of cervical cancer and screening among women in Addis Ababa.

Variable	Overall knowledge		COR 95% CI	AOR 95% CI
	Poor	Good		
**Educational level**				
No formal education	184 (53.6%)	159 (46.4%)	1.00	
Primary (1–8)	314 (48.6%)	332 (51.4%)	1.22 (0.94, 1.59)	1.23 (0.89, 1.68)
Secondary (9–12)	282 (50.6%)	275 (49.4%)	1.13 (0.86, 1.48)	1.10 (0.79, 1.52)
Diploma and above	111 (33.1%)	224 (66.9%)	2.34 (1.71, 3.19)	2.23 (1.49, 3.34)**
**Average monthly family income**				
≤ 3560 ETB (US $ 65 per month)	363 (49.9%)	364 (50.1%)		
3561 - 6050ETB (US$ 66–110 per months)	263 (46.2%)	306 (53.8%)	1.16 (0.93, 1.45)	0.93 (0.71, 1.22)
6051-11303 ETB(US $111–206 per month)	193 (54.8%)	159 (45.2%)	0.82 (0.64, 1.06)	0.68 (0.50, 0.93)*
> 11303 ETB(US $ 206 per month)	72 (30.9%).	161 (69.1%)	2.23 (1.63, 3.05)	1.5 (1.01, 2.23)**
**Knew health facility providing cervical cancer screening**				
No	354 (51.3%)	336 (48.7%)		
Yes	537 (45.1%)	654 (54.9%)	1.28 (1.06, 1.55)	1.34 (1.07, 1.68)*
**Know family members, friends or neighbors who have/had cervical cancer**				
No	937 (53.5%)	813 (46.5%)	1.000	
Yes	26 (19.8%)	105 (80.2%)	4.65 (3.00, 7.22)	5.45 (3.31, 8.97)**
**Source of information**				
Radio	19 (59.4%)	13 (40.6%)	1.000	
TV	346 (50.0%)	346 (50.0%)	1.46 (0.71, 3.01)	0.90 (0.39, 2.06)
Health Professionals	178 (40.2%)	265 (59.8%	2.18 (1.05, 4.52)	1.57 (0.67, 3.66)
Relatives & Friends	29 (51.8%)	27 (48.2%)	1.36 (0.57, 3.28)	0.92 (0.31, 2.73)
Health professionals and TV	153 (41.2%)	218 (58.8%)	2.08 (1.0, 4.34)	1.16 (0.49, 2.70)
All	65 (52.0%)	60 (48.0%)	1.35 (0.61, 2.97)	0.75 (0.30, 1.87)
Other***	6 (35.3%)	11 (64.7%)	2.68 (0.79, 9.07)	1.17 (0.3, 4.89)

* = P-Value <0.05, ** = P-Value <0.001, CI = Confidence Interval, TV = Television, ETB = Ethiopian Birr, COR = Crude Odds Ratio, AOR = Adjusted Odds Ratio, *** = social media like Facebook and telegram

## 4. Discussion

Based on a citywide survey of women aged 30–49 years, we found that factors such as higher educational attainment, high average monthly income, knowing of healthcare facilities offering cervical cancer screening, knowing someone diagnosed with cervical cancer, and source of information were significantly associated with overall knowledge of cervical cancer and screening.

Women with higher educational attainment were twice as likely to have greater overall good knowledge of cervical cancer and screening compared to those with lower educational attainment. This finding is similar to findings from studies conducted in Northwest Ethiopia (AOR = 2.18) [30], Jimma [27], South Africa [31], Ghana [32], and Tanzania (AOR = 2.59) [33]. However, it is much lower than the odds reported in Finote Selam, where women who completed university education had higher odds (AOR = 7.21) [34], Harar (AOR = 12.11) [35] as well as in Debre Tabor (AOR = 8.03) [36] and Nepal (AOR = 7.818) [37]. Across all these studies discussed above, higher educational attainment was strongly associated with knowledge of cervical cancer and screening. This may be attributed to the fact that, as women’s educational status improves, they become more capable of reading and understanding information about cervical cancer, which in turn increases their knowledge of cervical cancer and screening procedure.

A higher average monthly income was significantly associated with overall good knowledge of cervical cancer. This finding is supported by studies conducted in Dessie town [38] and South Africa [31]. However, it is opposite to the study conducted in Aria, West Wollega, where women with low income had higher knowledge [39] and in Debre Tabor, where women with high income had low knowledge [36]. The relationship between average monthly family income and knowledge of cervical cancer appears to vary across various contexts, indicating that income may not be the only factor influencing knowledge levels. This may be because of several factors. Women with higher incomes often have better access to information, higher education levels, and healthcare services, which enhances their knowledge of cervical cancer compared with women with lower incomes. However, conflicting findings from Aria, West Wollega, and Debre Tabor have challenged this assumption. In Aria, women with lower income exhibited higher levels of knowledge, which may be attributed to community-based health programs targeting lower-income populations. Grass root awareness initiatives, such as those conducted by NGOs or local health workers, may be more effective in these areas, directly engaging low-income women. The strong link between income and knowledge of cervical cancer emphasizes the need to tackle economic barriers in public health.

The findings of this study indicated that women who knew healthcare facilities offering cervical cancer screening had good overall knowledge of cervical cancer and screening. The results of this study are supported by a study conducted in Benishangul Gumz, Ethiopia [40]. These findings indicated that both awareness of and knowing healthcare facilities offering cervical cancer screening are crucial for enhancing women’s overall knowledge about cervical cancer and its screening.

Knowing someone having cervical cancer is associated with overall knowledge of cervical cancer and screening. This finding is supported by studies conducted in Jimma town [27], Durame, Ethiopia [41], Northwest Ethiopia, Finota Selam [34], Gurage zone [42], Nigeria [43], and Palestinian women [44]. The reason for similarity could be due to the fact that a woman who knows others suffering from cervical cancer is likely to be more familiar with its symptoms and related factors. Witnessing someone go through the illness may highlight the importance of recognizing the symptoms, severity, and critical importance of early detection. This in turn, motivates women to educate themselves about cervical cancer.

The strength of this study lies in the population-level data collected through face-to-face interviews conducted by female interviewers, which minimized misunderstandings of the questions. Although we did not obtain evidence to compare our findings with other population-based studies in similar settings, the results can be generalized to women aged 30–49 years in Addis Ababa.

## 5. Conclusion and recommendations

Nearly half of the women lacked awareness of cervical cancer and screening, as well as poor knowledge of risk factors and symptoms, resulting in an overall low level of knowledge among the study participants. Factors such as higher educational attainment, higher family income, knowledge of risk factors, knowing of healthcare facilities offering cervical cancer screening, and knowing someone who had cervical cancer significantly associated with the overall knowledge level. The findings of this study highlight the importance of empowering women through targeted and focused community-level health education. This is essential for increasing awareness and knowledge of cervical cancer and screening, as well as knowledge of risk factors, symptoms, and benefits of early detection and treatment.

## Supporting information

S1 File Inclusive questions, This file consists of questions helps to improve transparency in the reporting of research performed and outlines ethical, cultural, and scientific considerations specific to inclusivity in global research. This file describes ethical considerations, adherence to research protocol and study participants.
(DOCX)

S2 File STROBE Statement Checklist of items that should be included in reports of cross-sectional studies. It contains the title and abstract, Introduction, Objective, methods, results, discussions, limitations and other important descriptions.
(DOCX)
