# Supplementary material for: Factors affecting cervical cancer knowledge among women in Addis Ababa, Ethiopia: A population-based study
Source: PLOS Glob Public Health. 2025 Jul 28;5(7):e0004961. doi: 10.1371/journal.pgph.0004961 (PMC12303297; doi:10.1371/journal.pgph.0004961)
Supplement: S2 File — It contains the title and abstract, Introduction, Objective, methods, results, discussions, limitations and other important descriptions. (DOCX) [file pgph.0004961.s002.docx]

STROBE Statement—Checklist of items that should be included in reports of ***cross-sectional studies***

|  | Item No | Recommendation | Page No |
| --- | --- | --- | --- |
| **Title and abstract** | 1 | Factors affecting cervical cancer knowledge among women in Addis Ababa, Ethiopia: A population-based cross-sectional study | 1 |
|  |  | The objective of this study is to assess the level of CCa and screening awareness, knowledge, and predictors among women aged 30-49 years in Addis Ababa, Ethiopia. A total of 1980 women aged 30-49 were randomly selected through multistage sampling in Addis Ababa. Data were collected using the Kobo Toolbox after developing and validating the data collection tools. Data were analyzed using SPSS version 26. Logistic regression was used to identify predictors of cervical cancer awareness and knowledge, with a significance level set at a P-value of <0.05. Of the 1,890, 1,881 (99.5%) responded, 1,736 (92.3%) had heard of CCa, 1,015 (54.0%) were aware that Human Papillomavirus (HPV) infection is a risk factor, and 1,237 (65.8%) knew that HPV vaccination prevents CCa. More than half, 1,025 (54.5%) (95%CI=52.2%–56.8%) had good knowledge of risk factors, 980 (52.1%) (95% CI= 49.8, 54.4%) had poor knowledge of symptoms, and 963 (51.2%) (95% CI = 48.9%, 53.5%) exhibited poor awareness. Overall, 990 (52.6%) (95% CI = 50.4, 54.9%) participants had good knowledge of cervical cancer. Factors such as higher educational attainment (AOR=1.80; 95% CI: 1.19, 2.71), higher family income (AOR=1.69; 95%CI: 1.18, 2.40), greater knowledge of risk factors (AOR=11.25; 95% CI: 8.76, 14.45) Knowing Health facility offering screening (AOR=1.30; 95% CI: 1.02, 1.66) and knowing someone having cervical cancer (AOR=5.46; 95% CI: 3.31, 8.97) were significantly associated with the overall knowledge levels. Awareness and knowledge about cervical cancer and screening are suboptimal. These findings underscore the need for innovative strategies to enhance awareness and knowledge of cervical cancer to reduce the high burden of the disease in the city and other regions of Ethiopia. | 2 |
| Introduction | | | |
| Background/rationale | 2 | Cervical cancer is the second leading cause of morbidity and cancer death among women in Addis Ababa and other parts of Ethiopia[1, 2]. In Ethiopia, 8,168 new cases of cervical cancer are registered in 2023, indicating an incidence of 22.3 per 100,000 at standardized age. In the same year, 5,975 women died of the disease, corresponding to an age-standardized mortality rate of 16.8 per 100,000 women. Without intervention, a projected total of 191,876 women in Ethiopia will die from cervical cancer between 2020 and 2070[3, 4]. Nearly two-thirds of the cases occurring in Addis and other parts of the country are diagnosed at an advanced stage of the disease [5, 6] when treatment is less effective and the chance of survival is poor[6], due to low uptake of early screening and detection[7]. This figure, with two-third of cases occurring in Addis Ababa, is underestimated because the registry does not collect cancer cases from all hospitals and diagnostic facilities in the city, and many patients seek traditional rather than conventional medicine, leading to under diagnosis[8].  In low-middle income countries (LMICs), where resources are scarce and available treatment choices are sometimes inaccessible and expensive, cervical cancer continues to be a major worldwide burden and a major therapeutic challenge[9]. Therefore, it is crucial for all nations to endorse the 2020 World Health Assembly resolution calling for the "Elimination of Cervical Cancer" by 2030, with the following three goals: vaccinating 90% of girls against human papilloma virus HPV by age 15, screening 70% of women with high-performance tests at ages 35 and 45, and treating 90% of precancerous lesions and managing 90% of invasive cancer cases[10] .  Following the World Health Organization's cervical cancer elimination strategies, the Ethiopian government launched guidelines for cervical cancer elimination, focusing on raising awareness and knowledge among target women and the community as well as providing screening services and treatment[11].  Many studies have reported that most women have low awareness and knowledge. Studies from Addis Ababa showed that 43–76% of women had heard of cervical cancer screening (9-11). Less than one-third [12] of women were knowledgeable about cervical cancer . Studies from other parts of Ethiopia, such as Butajera, indicated that only 36% of women were aware of cervical cancer  and less than 5% knew the symptoms of cervical cancer [13]. In Assosa, 53.5% of the participants demonstrated good knowledge of cervical cancer.[14] Studies involving women visiting health institutions for various services also showed a low level of awareness and knowledge. For example, among women visiting primary health care centers for antenatal follow-up, family planning, or postnatal care, 42.7% had heard of cervical cancer screening and 27.7% had adequate knowledge of cervical cancer screening.[14] Similarly, a community-based cross-sectional study of parents or guardians of daughters aged 9–17 years in Akaki-Kalty sub-city reported that 41.7% and 72.0% of women had good knowledge of cervical cancer and HPV, respectively,[15] while 27.0% of women had never heard of the HPV vaccine[15].  However, these studies were limited because they were based on clinical series, small geographic areas, or women living with HIV, and their findings cannot be generalized to all women in Addis Ababa.  In this study, we examined the knowledge and awareness of cervical cancer risk factors and preventive measures among women aged 30-49 in Addis Ababa, based on data from a city-wide representative survey. We focused on this age group because they are recommended to undergo cervical cancer screening by the WHO and may be raising children who are eligible for the HPV vaccine. | 3-4 |
| Objectives | 3 | The aim of this study is to assess the awareness and knowledge and factors affecting knowledge of cervical cancer and screening | 4 |
| Methods | | | |
| Study design | 4 | A population-based cross-sectional study design was used | 5 |
| Setting | 5 | Addis Ababa, the capital city of Ethiopia, has a population of over 5.7 million.[16] The city is divided into 11 administrative sub-cities, comprising 111 districts and 5,536 enumeration areas (EAs). There are 99 public primary care health centers and 12 public hospitals in Addis Ababa.  This study included women aged 30-49 who had lived in Addis Ababa for more than six months and had no history of hysterectomy. It is estimated that nearly 17% of women are of reproductive age, and more than one-third 40% of women are between 30 and 49 years old.[17] Cervical cancer screening services are provided in all public health institutions | 4 |
| Participants | 6 | The sample size was determined using the WHO-STEP sample size determination formula for non-communicable diseases (NCDs),[18] with a design effect of 2.0, an age-sex estimation of 2 for females only, an 80% response rate, and a 5% margin of error. The proportion of 56.3% with a lack of knowledge about cervical cancer  screening in Addis Ababa, as reported by Getachew et al. (2019),[14] was also considered. Based on these assumptions, a sample size of 1890 was obtained. The number of enumeration areas (EA) or clusters (K) was estimated by dividing the sample size (1890) by the number of households (30 HHs) interviewed per cluster. Therefore, the number of clusters (K) was calculated as 1890/30 = 63. Each cluster represents an EA.  Multistage sampling techniques were employed in this study. In the first stage, the city was clustered into 11 sub-cities. In the second stage, 63 EAs were proportionally allocated to the sub-cities using computer-based proportional allocation of the sample size. In the third stage, 30 HHs were randomly selected for each EA. Each EA was delineated from four directions (North, South, East, and West), and households were numbered and counted. The interval was calculated by dividing the number of households by 30 (K = No. of HHs/30). The k^th^ interval varied among EAs based on household size. If there were no target women in the selected HHs, the immediate HHs was chosen and the interval was continued. In cases in which there was more than one target woman in the HHs unit, one woman was randomly selected from the data collector. Three repeated visits were conducted to include all the randomly selected women during the interviews. Of the 1890 women selected to participate in the study, eight could not be traced, and one declined to participate.  Women aged 30-49 years were included in this study based on the WHO's recommendation for cervical cancer screening in low-income countries using the “See and Treat” approach [10]. Women younger than 30 years and women older than 49 years were excluded because they were not eligible for cervical cancer screening in the general population.[10] Women who were pregnant or within three months postpartum were excluded based on guidelines for cervical cancer prevention and control in Ethiopia.[19] Women with a history of total hysterectomy were also excluded from the study | 5 |
| Variables | 7 | **Outcome variable:** knowledge level (Coded as overall knowledge level; Poor/Good)  **Independent variables**: it includes socio-demographic variables such as age, educational level, current occupational and marital status, partner`s educational status and average family monthly income. Variables for assessing awareness level includes hearing about cervical cancer and screening, knowing someone contracting cervical cancer, knowing cervical cancer screening availability and frequencies of screening, knowing health institution offering screening knowing about HPV vaccination and prevention methods of cervical cancer. Variables for knowledge of risk factors include knowing about HPV infection, HIV and other STIs infection, multiple partners, non-condom use, high parity, long term uses of contraceptives, initiation of sex at young age, smoking, etc. Variables for symptoms of the disease, such as Vaginal bleeding between menstrual periods, a persistent smelly vaginal discharge, Discomfort or pain during sex, longer and heavy menstrual periods, Vaginal bleeding after menopause, Persistent lower abdominal/pelvic pain, Vaginal bleeding during or after sex, Unexplained weight loss could be S/S of cervical cancer and Vaginal itching were used to collect data. |  |
| Data sources/ measurement | 8* | Individual participants were the data sources, interviewer administered questionnaires were used to collect the data |  |
| Bias | 9 | Data was collected using Kobo Toolbox software, which made all questions mandatory to prevent missing data, and were checked daily for completeness and consistency. |  |
| Study size | 10 | WHO-STEP sample size determination formula for non-communicable diseases (NCDs), with a design effect of 2.0, an age-sex estimation of 2 for females only, an 80% response rate, and a 5% margin of error. The proportion of 56.3% with a lack of knowledge about cervical cancer  screening in Addis Ababa, as reported by Getachew et al. (2019),[14] was also considered. Based on these assumptions, a sample size of 1890 was obtained. The number of enumeration areas (EA) or clusters (K) was estimated by dividing the sample size (1890) by the number of households (30 HHs) interviewed per cluster. Therefore, the number of clusters (K) was calculated as 1890/30 = 63. Each cluster represents an EA.  Multi-stage sampling was used. In the first stage, the city was clustered into 11 sub-cities. In the second stage, 63 EAs were proportionally allocated to the sub-cities using computer-based proportional allocation of the sample size. In the third stage, 30 HHs were randomly selected for each EA. Each EA was delineated from four directions (North, South, East, and West), and households were numbered and counted. The interval was calculated by dividing the number of households by 30 (K = No. of HHs/30). The k^th^ interval varied among EAs based on household size. If there were no target women in the selected HHs, the immediate HHs was chosen and the interval was continued. In cases in which there was more than one target woman in the HHs unit, one woman was randomly selected from the data collector. Three repeated visits were conducted to include all the randomly selected women during the interviews. Of the 1890 women selected to participate in the study, eight could not be traced, and one declined to participate. | 5 |
| Quantitative variables | 11 | Data was collected using Kobo Toolbox software, which made all questions mandatory to prevent missing data, and were checked daily for completeness and consistency.  Awareness and knowledge of cervical cancer symptoms were measured using 10 separate questions, and participants with values above the mean were considered to have good awareness and knowledge of cervical cancer symptoms. Knowledge of risk factors was assessed using 13 questions. Those scoring above the mean were categorized as having good knowledge of risk factors, while those scoring above the mean were categorized as having poor knowledge. The overall knowledge level of cervical cancer was assessed using the mean value of the knowledge of symptoms and risk factors. Participants scoring above the mean were considered to have good knowledge, whereas those scoring below the mean were considered to have poor knowledge of cervical cancer. | 7 |
| Statistical methods | 12 | (*a*) Data were checked for normal distribution using the Kolmogorov-Smirnov test (P<0.001, indicating non-normal distribution), low skewness with Pearson's coefficient of skewness (0.86 = indicating slight right-skew), and for collinearity and goodness of fit using the Variance Inflation Factor (VIF < 10 as the cutoff point) and the Chi-square test.  Binary and multivariate logistic regression analyses were conducted using backward logistic regression, which excluded variables with no or small effects from the final model. Variables with P-values <0.25 during univariable logistic regression as well as important variables were selected for multivariable logistic regression to control for confounders [27, 28]. The final model of multivariable logistic regression was used to declare the association of variables using adjusted odds ratios with 95% confidence intervals, and P-values <0.05 were used to indicate the association between independent and dependent variables. | 7 |
|  |  | (*b*) Describe any methods used to examine subgroups and interactions  - Not applicable | 7 |
|  |  | (*c*) Explain how missing data were addressed  _ missed variable are coded as missing (999) and excluded from analysis |  |
|  |  | (*d*) If applicable, describe analytical methods taking account of sampling strategy  - Not applicable |  |
|  |  | (*e*) Describe any sensitivity analyses  - Not applicable |  |
| Results | | | |
| Participants | 13* | Almost all 1,881 participants (99.5%) responded to the survey and analysed | 8 |
|  |  | (b) not available during data collection, Three repeated visits were conducted to get the selected participants but lost. |  |
|  |  | (c) Consider use of a flow diagram  - NA |  |
| Descriptive data | 14* | The median age of the participants was 35 (IQR, 8) years. Regarding educational attainment of the study participants, 646 (34.3%) attained primary-level education. More than half 1064 (56.6%) of the participants were housewives (stay-at-home mothers), and 1,411 (75.0%) were married. Over one-third of the 727 (38.6%) had an average monthly income of 3,560 ETB, equivalent to US$ 65/month | 8 |
|  |  | (b) no missing value was observed except as result of skip patterns |  |
| Outcome data | 15* | Nearly half of study participants (52.6%; 95% CI = 50.4, 54.9%) demonstrated good overall knowledge |  |
| Main results | 16 | Women with a diploma or higher education level had 1.8 times higher odds (AOR=1.80; 95% CI: 1.19, 2.71) of having good overall knowledge of cervical cancer and screening than women with no formal education. As the average monthly income increased, the knowledge of women also increased. The odds of having good overall knowledge was 1.69 time higher (AOR=1.69; 95%CI: 1.18, 2.40) among women with higher income than among those with low income. The odds of having good overall knowledge among women with good knowledge of risk factors was more than 11 times higher (AOR=11.25; 95% CI: 8.76, 14.45) compared among those with poor knowledge. Knowledge of health facilities offering cervical cancer screening is also significantly associated with knowledge of cervical cancer and screening. The odds of having good overall knowledge was 1.3 times higher (AOR=1.30; 95% CI: 1.02, 1.66) among women who knew health facilities providing cervical cancer screening. Likewise, the odds of having good overall knowledge of cervical cancer and screening were more than five times higher (AOR=5.46; 95% CI: 3.31, 8.97) among women who knew someone with cervical cancer than among their counterparts. The odds of having good overall knowledge of cervical cancer and screening were more than twice (AOR=2.32; 95% CI: 1.09, 4.94) among women who obtained information from health professionals and television compared to those who obtained information from the radio, and more than three times higher (AOR=3.62; 95% CI: 1.69, 7.77) among women who obtained information from multiple health sources compared to those who obtained information from the radio. | 14 |
|  |  | (*b*) Report category boundaries when continuous variables were categorized  - NA |  |
|  |  | (*c*) If relevant, consider translating estimates of relative risk into absolute risk for a meaningful time period  - NA |  |
| Other analyses | 17 | Report other analyses done—eg analyses of subgroups and interactions, and sensitivity analyses   - NA |  |
| Discussion | | | |
| Key results | 18 | Based on a citywide survey of women aged 30-49 years, we found that factors such as higher educational attainment, high average monthly income, high knowledge of risk factors, knowledge of healthcare facilities offering cervical cancer screening, knowing someone diagnosed with cervical cancer, and source of information were significantly associated with overall knowledge of cervical cancer and screening.  Women with higher educational attainment were twice as likely to have greater overall good knowledge of cervical cancer and screening as those with lower educational attainment. This finding is similar to findings from studies conducted in Northwest Ethiopia (AOR=2.18)[29], Jimma[26], South Africa[30], Ghana[31], and Tanzania (AOR=2.59)[32], but much lower than those reported in Finote Selam, where women who completed university education had higher odds (AOR=7.21)[33], Harar (AOR=12.11)[34] as well as in Debre Tabor (AOR=8.03)[35] and Nepal (AOR=7.818)[36]. In all the studies discussed above, higher educational attainment was strongly associated with knowledge of cervical cancer and screening. This could be attributed to the fact that, as women's educational status improves, they become better able to read and understand information about cervical cancer, which in turn increases their knowledge of cervical cancer and screening.  A higher average monthly income was significantly associated with overall good knowledge of cervical cancer. This finding is supported by studies conducted in Dessie town[37] and South Africa[30]. However, it is opposite to the study conducted in Aria, West Wollega, where women with low income had higher knowledge [38] and in Debre Tabor, where women with high income had low knowledge.[35] The relationship between average monthly family income and knowledge of cervical cancer appears to vary across various contexts, indicating that income may not be the only factor influencing knowledge levels. This may be because of several factors. Women with higher incomes often have better access to information, higher education levels, and healthcare services, which enhances their knowledge of cervical cancer compared with women with lower incomes. However, conflicting findings from Aria, West Wollega, and Debre Tabor have challenged this assumption. In Aria, women with lower income exhibited higher levels of knowledge, which may be attributed to community-based health programs targeting lower-income populations. Grassroot awareness initiatives, such as those conducted by NGOs or local health workers, may be more effective in these areas, directly engaging low-income women. The strong link between income and knowledge of cervical cancer emphasizes the need to tackle economic barriers in public health.  We identified a strong association between women’s overall knowledge of cervical cancer, screening, and knowledge of risk factors. However, we were unable to obtain conclusive evidence to support or disprove this finding. The lack of data to validate or invalidate the results of this study suggests potential gaps in the existing research. The significant relationship observed in this study suggests that educational efforts focusing on risk factors could significantly improve overall knowledge of cervical cancer.  The findings of this study indicated that women who were aware of healthcare facilities offering cervical cancer screening had good overall knowledge of cervical cancer and screening. The results of this study are supported by a study conducted in Benishangul Gumz, Ethiopia[39]. These findings indicated that both awareness of and access to healthcare facilities offering cervical cancer screening are crucial for enhancing women's overall knowledge about cervical cancer and its screening.  Knowing about cervical cancer is associated with overall knowledge of cervical cancer and screening. This finding is supported by studies conducted in Jimma town,[26] Durame, Ethiopia,[40] Northwest Ethiopia, Finota Selam,[33] Gurage zone,[41] Nigeria,[42] and Palestinian women,[43] The similarity may be due to the fact that a woman who knows others suffering from cervical cancer is likely to be more familiar with its symptoms and related factors. This awareness, stemming from witnessing someone enduring the disease, emphasizes the symptoms, severity, and critical importance of early detection. As a result, it encourages women to educate themselves on cervical cancer.  Obtaining information from various sources was significantly associated with women’s knowledge. This study found that women who received information from health professionals were more likely to be knowledgeable. This finding is supported by the studies conducted in the Wolita zone, Southern Ethiopia,[44] Eastern Ethiopia,[34] in Gonder town,[45] and Harar.[34] On the other hand, this study also showed that information from multiple sources is significantly associated with knowledge levels compared to information from a single source. Utilizing both health professionals and the media is important for raising awareness of cervical cancer and screening among women. As women's knowledge about cervical cancer and screening increases, the uptake of cervical cancer screening will also increase | 15 -17 |
| Limitations | 19 | Lack of similar study in the study area to compare the finding |  |
| Interpretation | 20 | Nearly half of the women lacked awareness of cervical cancer and screening, as well as poor knowledge of risk factors and symptoms, resulting in an overall low level of knowledge among the study participants. Factors such as higher educational attainment, higher family income, knowledge of risk factors, knowledge of healthcare facilities offering cervical cancer screening, knowing someone who had cervical cancer, and the source of information were significantly associated with the overall knowledge level. The findings of this study highlight the importance of empowering women through targeted and focused community-level health education. This is essential for increasing awareness and knowledge of cervical cancer and screening, as well as knowledge of risk factors, symptoms, and benefits of early detection and treatment. | 18 |
| Generalisability | 21 | The strength of this study lies in the population-level data collected through face-to-face interviews conducted by female interviewers, which minimized misunderstandings of the questions. The samples were randomly assigned to each sub-cities to get representativeness. Multistage sampling was used.  Therefore, the results can be generalized to women aged 30-49 years in Addis Ababa. |  |
| Other information | | | |
| Funding | 22 | No fund received |  |

*Give information separately for exposed and unexposed groups.

**Note:** An Explanation and Elaboration article discusses each checklist item and gives methodological background and published examples of transparent reporting. The STROBE checklist is best used in conjunction with this article (freely available on the Web sites of PLoS Medicine at http://www.plosmedicine.org/, Annals of Internal Medicine at http://www.annals.org/, and Epidemiology at http://www.epidem.com/). Information on the STROBE Initiative is available at www.strobe-statement.org.
